# Physical equivalency of wild type and galactose α 1,3 galactose free porcine pericardium; a new source material for bioprosthetic heart valves

**DOI:** 10.1016/j.actbio.2016.06.007

**Published:** 2016-09-01

**Authors:** Christopher McGregor, Guerard Byrne, Benyamin Rahmani, Elisa Chisari, Konstantina Kyriakopoulou, Gaetano Burriesci

**Affiliations:** a University College London, Institute of Cardiovascular Science, United Kingdom; b University College London, Department of Mechanical Engineering, United Kingdom

**Keywords:** Bioprosthetic heart valve, Xenogeneic antigens, Gal knockout, Tissue equivalency

## Abstract

Humans make high levels of antibody to carbohydrates with terminal galactose α 1,3 galactose (Gal) modifications. This Gal antigen is widely expressed in other mammals and is present on an array of current animal derived biomedical devices including bioprosthetic heart valves. There is growing interest in using Gal-free animal tissues from Gal knockout pigs (GTKO) as these tissues would not be affected by anti-Gal antibody mediated injury. In this study we compare the composition and biophysical characteristics of glutaraldehyde fixed porcine pericardium from standard and GTKO pigs. We show that with the exception of the Gal antigen which is only present in standard pig tissue both GTKO and standard pig tissue have the same general morphology and collagen content. Moreover uniaxial stress testing and suture retention testing indicate the tissues are equivalent in tensile strength. These studies indicate that genetic disruption of the α-galactosyltransferase (GGTA-1) which blocks synthesis of the Gal antigen has no significant impact on the structural integrity of porcine pericardium and suggest that this tissue could be directly substituted for standard pig pericardium in biomedical devices such as bioprosthetic heart valves.

**Statement of Significance:**

Surgical heart valve replacement is a proven life saving therapy to treat heart valve dysfunction due to birth defects, infection and the effects of aging. Bioprosthetic heart valves (BHV) made from glutaraldehyde fixed animal tissues are an effective durable therapy in older patients (>60 years) but exhibit age-dependent structural valve degeneration (SVD) in younger patients (<60 years). SVD is principally caused by BHV calcification. Immune injury contributes to age-dependent SVD through the interaction of galactose α 1,3 galactose (Gal) a dominant xenogeneic antigen present on commercial BHVs and universally abundant human anti-Gal antibody. This study measures the tissue equivalency between standard pig pericardium and Gal-free pericardium from genetically modified pigs as a first step towards making Gal-free BHVs.

## Graphical abstract

**Figure:**
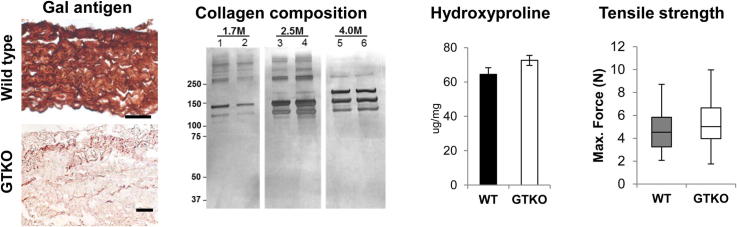

## 1 Introduction

Bioprosthetic heart valves (BHVs) have been produced from bovine and porcine tissues for over 50 years. These devices have been very successful as valve replacement therapy to treat heart valve disease and recent trends show a significant shift towards their use compared to mechanical heart valves (MHVs) [1]. BHVs have better hemodynamic properties compared to MHVs and generally do not require chronic anticoagulant therapy which is necessary for MHVs [2]. Nevertheless, despite more than half a century of extensive scientific and clinical investigations, current BHVs remain associated with limited durability due to structural valve degeneration (SVD) caused by dystrophic calcification, inflammation, and leaflet tearing resulting in failure of the prosthesis. The frequency of SVD is not constant but is strongly affected by patient age [3]. At 15 years post-surgery 60–70% of older patients (>75 years) have freedom from SVD. This falls to 55% in patients aged 60–75 years. Up to 100% of BHVs fail within 5 years in patients <35 years of age [4].

Clinical BHVs are based on glutaraldehyde fixation of bovine or porcine pericardium or the use of porcine heart valves. This basic technology has been augmented with various post fixation anti-calcification processing strategies to reduce dystrophic BHV calcification [5], [6]. These anti-calcification processes are commonly designed to extract phospholipids which are thought to act as a nidus for calcium binding. Testing in juvenile animal models shows significant reduction of BHV calcification after anti-calcification treatments [5], [6], [7], [8], [9], [10] but there is scant evidence for a clinical improvement in BHV durability or prevention of age-dependent SVD. This suggest that calcification in juvenile animal models primarily results from passive, phospholipid-dependent, calcification but that in patients additional mechanism(s) contribute to calcific BHV degeneration, particularly to age dependent SVD.

SVD has long been associated with an inflammatory response [2]. Recent studies of BHVs derived from left ventricular assist devices show early fibrin deposition, microthrombi formation, and macrophage, antibody, and complement infiltration into the BHV matrix after limited implantation in patients and prior to evidence of calcification, infection or pannus growth [2], [11], [12], [13]. This pathology is consistent with an antibody mediated BHV immune injury which can promote tissue calcification [14]. We have recently demonstrated that antibody universally present in high quantities in humans to the xenogeneic carbohydrate galactose α 1,3 galactose (Gal) accelerates calcification of glutaraldehyde fixed porcine pericardium but does not increase calcification of Gal-free pericardium derived from pigs with a mutation in the GGTA-1 glycosyltransferase (GTKO pigs) required for synthesis of the Gal antigen [15]. This anti-Gal antibody induced calcification occurs even after the tissues are treated with phospholipid extracting anti-calcification processing confirming that immune injury can independently affect tissue calcification [16].

The Gal antigen is widely expressed in porcine and bovine tissue and is retained in currently used commercial BHVs [15], [17]. Unlike protein antigens Gal remains immunogenic after fixation and is reported to induce a transient induction of anti-Gal antibody in adult patients [17], [18] and a stronger sustained induction of anti-Gal antibody in children [19] after BHV replacement surgery. In non-human primates, the only other class of mammals which produce anti-Gal antibody, mitral valve replacement using commercially prepared wild type and GTKO porcine BHVs resulted in persistent and significantly elevated anti-Gal IgG level in WT BHV recipients but not in GTKO BHV recipients [20]. The combination of anti-Gal antibody in patients, and the high level of Gal antigen on commercial BHVs enables an immediate and chronic innate antibody dependent inflammation of the valve without the need for a specific immune induction which likely contributes to age-dependent SVD. These results suggest that BHVs produced from GTKO pig tissues and lacking the Gal antigen would be resistant to anti-Gal induced calcification and may help to prevent age-dependent SVD. This study tests the physical equivalence of wild type and GTKO porcine pericardium as an essential first step towards developing Gal-free GTKO BHVs.

## 2 Materials and methods

### 2.1 Pig tissue processing and fixation

Genetically modified GGTA-1 deficient (GTKO) pigs were produced in accordance with the Animals (Scientific Procedures) Act of 1986, published by the United Kingdom Home Office, and the Guide for the Care and Use of Laboratory Animals from the U.S. National Institute of Health (Publication No. 85-23, revised 1996) Conventional wild type (WT) pig tissues were obtained from a local abattoir. Heart lung blocks were collected from GTKO or wild type animals and chilled on ice prior to isolating the pericardium. Tissues were rinsed with water and dissected to recover the pericardial sac. The pericardial sac was rinsed in sterile saline (4 °C) split and cleaned of fat, rinsed extensively in sterile saline and stored overnight at 4 °C. Fresh pericardial samples were blotted dry and frozen at −80 °C. For fixation all tissues were fixed in 0.6% glutaraldehyde in 20 mM HEPES-Saline (pH 7.4) containing 13 mM MgCl_2_-H_2_O for 24 h at 4 °C, rinsed in sterile saline and stored in the same buffer containing 0.2% glutaraldehyde at 4 °C until used.

### 2.2 Hydroxyproline

The hydroxyproline content of fixed WT and GTKO pericardium was measured using dimethylamino-benzaldehyde (DMAB) after oxidation with Chloramine T. Pericardium (1 mg wet weight) was hydrolysed in 200 μl 6 N HCl at 120 °C for 3 h. Hydrolysed samples (10–50 μl) were transferred to 96-well plates and dried at 60 °C then incubated with 100 μl of Chloramine T for 5 min at room temperature followed by a 90 min incubation at 60 °C with 100 μl of DMAB reagent as described by the manufacturer (Sigma Aldrich, St Louis, MO, USA). The absorbance at 560 nm was measured using a Genios plate reader (Tecan UK Ltd, Reading, UK). Hydroxyproline content of pericardium was determined by reference to a standard curve.

### 2.3 Protein extraction, Gel electrophoresis and lectin blotting

Detergent soluble proteins were extracted from fresh split pig pericardium by incubating the tissue (200 mg/ml) in 2× Laemmli buffer (60 mM Tris pH 6.8, 2% SDS, 72 mM BME) at 4 °C for 24 h. Samples were centrifuged at 20,000×*g* for 30 min at 4 °C and stored at 4 °C. Collagen was isolated after acetic acid and pepsin digestion and differential NaCl precipitation was used to characterize the collagen content essentially as described [21]. In brief, fresh frozen pericardium was pulverized in liquid nitrogen, suspended (5 mg/ml) in 2 M NaCl, 50 mM Tris, 1 mM EDTA (pH 7.4) containing freshly added 1 mM phenylmethanesulfonylfluoride (PMSF) and N-ethylmaleimide (NEM) and incubated at 4 °C for 24 h with constant agitation. Samples were centrifuged at 30,000×*g* for 40 min at 4 °C and the pellet was incubated at 4 °C overnight in 0.5 M acetic acid (pH 2.5). The sample was centrifuged and the pellet resuspended in fresh acetic acid containing pepsin (Sigma Aldrich, St Louis, MO, USA) at a 1:10 pepsin:tissue ratio and incubated at 4 °C for 48 h. High molecular weight protein aggregates were removed from the pepsin extract by precipitation with 0.6 M NaCl 4 °C for 2 h and centrifugation. Crude collagen was recovered by precipitation with 2 M NaCl at 4 °C for 12 h. This pellet was dissolved in 50 mM Tris pH 7.4 and re-precipitated with 4 M NaCl to make a final total collagen extract. For differential collagen precipitation the crude collagen pellet was dissolved in 0.5 M acetic acid and dialysed against 0.4 M NaCl, 100 mM Tris (pH 7.4). Differential collagen precipitation was achieved by sequential dialysing against 1.7, 2.5 and 4.0 M NaCl, 100 mM Tris (pH 7.4). At each NaCl concentration the samples were centrifuged and the recovered pellets were dissolved in 50 mM Tris, 50 mM NaCl (pH 8) for analysis.

Progressive collagen precipitates were analysed by gel electrophoresis using a 7.5% Mini-PROTEAN acrylamide gels under non-reducing conditions (BioRad, Hercules, CA, USA). Separated proteins were detected by silver staining (Invitrogen, Carlsbad, CA, USA).

For detection of the Gal antigen protein extracts were separated on a 7.5% apparatus (7.5% Mini-PROTEAN® TGX™ Gel, BioRad) under reducing conditions and transferred to a PVDF membrane using a BioRad Mini-Trans Blot Module for 2 h at 90 V. Membranes were blocked with Dulbecco’s PBS containing 2% BSA and 0.3% Tween-20 for 2 h at ambient temperature. Gal antigen was detected using 3 μg/ml biotin-conjugated *Griffonia simplicifolia* isolectin B4 (GSIB-4, Vector Laboratories, Burlingame CA) diluted in blocking buffer at 4 °C overnight as recommended by the manufacturer. Membranes were washed in PBS containing 0.3% Tween-20 and GSIB-4 binding was detected by incubating with 1 μg/ml horseradish peroxidase (HRP) conjugated streptavidin (Pierce Streptavidin Poly-HRP, Thermo Scientific) for 2 h at room temperature. Streptavidin binding was observed with chemiluminescence (Pierce™ ECL Western Blotting Substrate, Thermo Scientific, Rockford, IL, USA) and recorded with a myECL Imager (Fisher Scientific UK Ltd, Loughborough, UK).

### 2.4 Histology

Fresh and glutaraldehyde fixed pericardial samples were embedded in Optimal Cutting Temperature material and cryosectioned (8 μm). Sections were air dried, fixed with acetone for 10 min and stored at −80 °C until used. Fresh and glutaraldehyde fixed samples were also fixed in formalin and paraffin embed for Masson’s Trichrome histology staining. Tissue sections were stained to detect the Gal antigen and collagens. Fresh and glutaraldehyde fixed tissues were stained with biotin-conjugated GSIB-4 (3 μg/ml) and murine monoclonal antibodies (2 μg/ml) to Collagen I (Anti-Collagen Type I, Sigma-Aldrich), III (Anti-Collagen Type III, Abcam, Cambridge, UK), and V (Anti-Collagen Type V, Abcam). Lectin and antibody solutions were produced in dPBS with 1% BSA and primary incubations were at 4 °C for 12 h. GSIB-4 binding was detected with HRP-conjugated streptavidin (Sigma-Aldrich) and mouse anti-collagen antibody binding was detected with biotin conjugated goat anti-mouse IgG (Sigma-Aldrich) and HRP-conjugated streptavidin. All sections were incubated with 3,3′-Diaminobenzidine (Sigma-Aldrich) and counter stained with haematoxylin.

### 2.5 Uniaxial tensile mechanical test

Dumbbell-shape test pericardial specimens were cut to 12 mm gauge length and 2 mm width, according to type 4 test specimen dimensions recommended by ISO 37-2011 [22]. Care was taken to cut the specimen in such a way to align the direction of the extracellular matrix (ECM) fibers at a 45° angle relative to the axis of the sample. The thickness of each specimen was measured from three equally spaced locations along the specimen length using a Mitutoyo gauge (Mitutoyo Corporation, Tokyo, Japan) immediately prior to testing. Uniaxial tensile test was performed for 32 samples of each tissue group using a Zwickyline tensile test machine (Zwick.Roell GmbH & Co, Ulm, Germany) equipped with a 2.5 kN load cell, a media container with temperature control unit and test Xpert software. Samples were submerged in 37 °C saline and loaded to failure applying a cross-head displacement rate of 20 mm/min. The maximum tensile force and strength, as well as the elongation at maximum force were obtained. Tensile strength (σ_u_, expressed in megapascals) was calculated by normalising the maximum tensile force (F, expressed in Newtons) by unit area of the unloaded cross-section of the specimen. Elongation at maximum force was determined as the percentage of increase in length at the maximum force F, with respect to the original length of the gauge.

### 2.6 Suture retention test

Suture retention tests were performed by pulling a suture from the pericardial tissue sample to determine the force necessary to tear the tissue. The test protocol was adopted from ANSI/AAMI/ISO7198 [23]. WT (n = 15) and GTKO (n = 15) tissues were cut in 1 × 2 cm specimens and a multi-filament suture (Ethicon VICRYL 5-0) was inserted 2 mm from the edge of the sample. Tests were performed on the Zwickyline tensile test machine, pulling the suture at a speed of 50 mm/min. The maximum of the force required to pull the suture through the sample was recorded.

### 2.7 Statistical analysis

All stress measurements were analysed using Microsoft Excel or GraphPad Prism software (GraphPad, San Diego, CA) and presented as means ± standard deviation. Where required, an unpaired two-tailed *t*-test with Welch’s correction was used to compare two population means, and a P-value of <0.05 was considered significant.

## 3 Results

### 3.1 Composition of Porcine WT and GTKO Pericardium

**Fig. 1:**
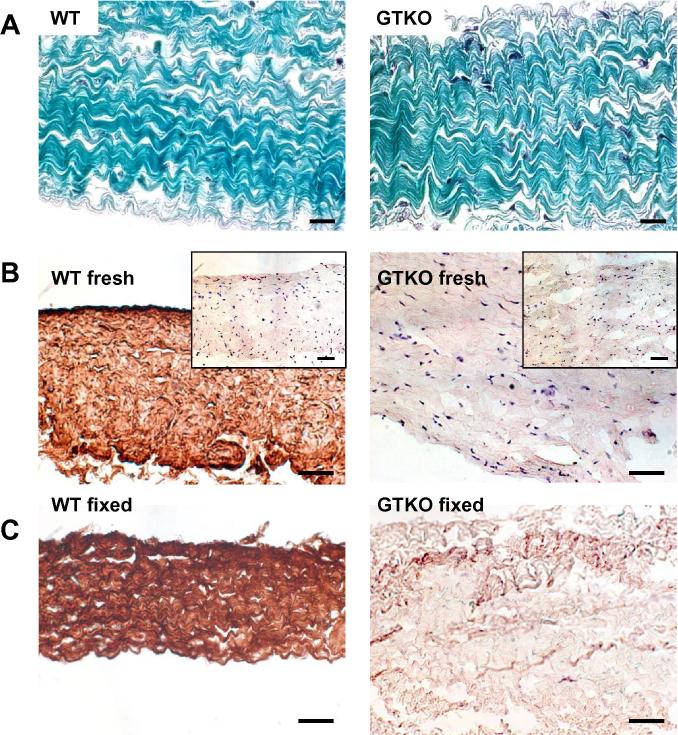
Morphology of WT and GTKO pig pericardium. A. Trichrome stain. B and C. Immunohistochemistry using the lectin GSIB-4 to detect the Gal antigen in fresh (B) and 0.6% glutaraldehyde fixed (C) pericardium. Inserts in B show Gal-specific inhibition of lectin binding by 10 mM Gal trisaccharide. Scale bars are 20 μm in A, and 50 μm in B and C.

Split porcine pericardium is largely a matrix of extracellular protein with strong trichrome collagen staining present in both wild type (WT) and GTKO pericardium (Fig. 1). GSIB-4 lectin staining of fresh and glutaraldehyde fixed WT porcine pericardium detects abundant Gal antigen expression (Fig. 1B and C). Soluble competition using 10 mM Gal-trisaccharide eliminates the bulk of GSIB-4 staining to WT tissue (insert Fig. 1B) and demonstrates the specificity of lectin staining. There is no Gal expression evident in fresh or fixed GTKO porcine pericardium (Fig. 1B and C).

**Fig. 2:**
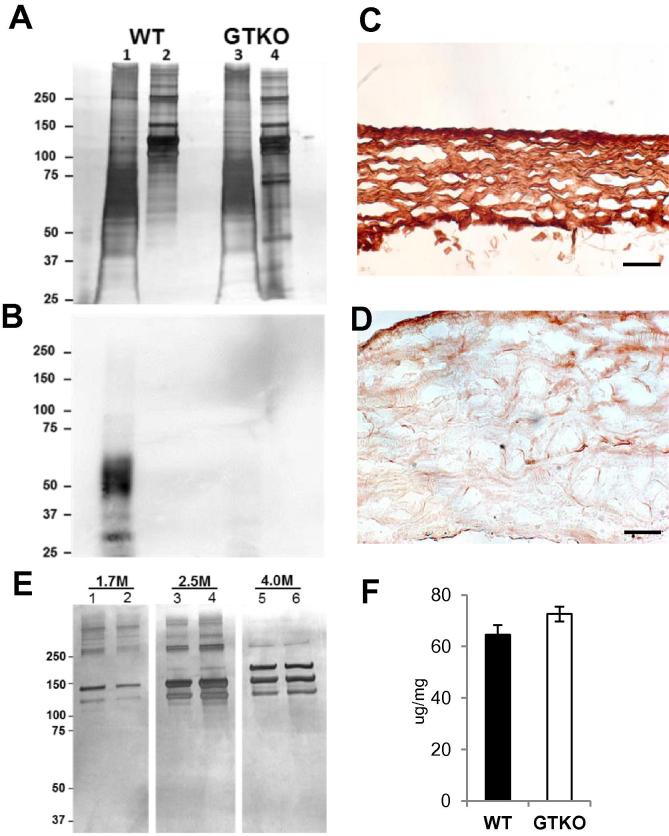
Pericardial composition and hydroxyproline content. A. Silver stained polyacrylamide electrophoresis of detergent soluble (lanes 1 and 3) and total pepsin treated collagen extracts (lanes 2 and 4) from WT and GTKO pig pericardium. Both tissue sources show very similar profiles of extracted proteins. B. Western blot of the gel in A using the Gal-specific lectin GSIB-4. Gal bearing glycoproteins are evident only in proteins from the WT detergent extract. C. Fixed WT pericardium extracted with Laemmli buffer and stained with GSIB-4. Fixation traps detergent soluble proteins and retains Gal expression. D. Fresh WT pericardium extracted with Laemmli buffer and stained with GSIB-4. Gal is largely depleted by Laemmli extraction from fresh pericardium as indicated by the lectin blot in B. E. Differential collagen precipitation from acetic acid pepsin digests of WT (lanes 1, 3 and 5) and GTKO (lanes 2, 4 and 6) pericardium. The silver stained polyacrylamide gel shows pepsin treated collagen extracts solubilised at 1.7, 2.5 and 4 M NaCl concentrations. Both tissue show the same collagen composition. F. Hydroxyproline content of WT (n = 16) and GTKO (n = 16) pericardial samples. Hydroxyproline is a common modified amino acid present in collagen and a useful estimate of collagen content. Error bars are standard error of the mean. There is no difference (Student’s *t*-test p > 0.05) between the samples. Scale bars in C and D are 50 μm.

**Fig. 3:**
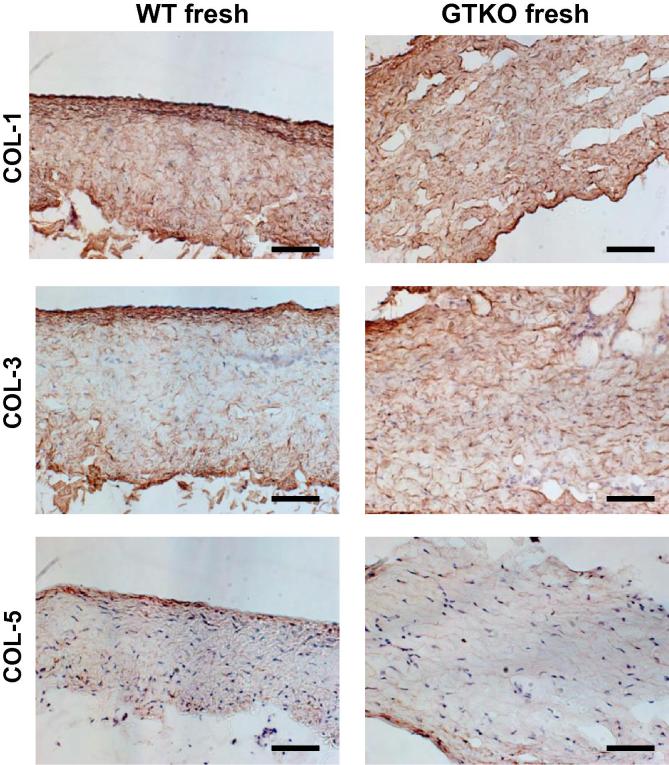
Immunohistology of collagen expression in WT and GTKO pig pericardium. Fresh pericardium was stained with antibody to collagen I, III and V. Scale bars are 50 μm.

Detergent soluble proteins and total collagen extracts from WT and GTKO show similar protein profiles (Fig. 2A). GSIB-4 lectin blotting of these extracts detects Gal antigen in WT detergent soluble pericardial proteins but not in WT collagen or in GTKO detergent soluble proteins or collagen extracts (Fig. 2B). Fixation of WT pericardium prior to Laemmli buffer extraction appears to trap soluble proteins and retains the Gal antigen (Fig. 2C) whereas Laemmli extraction of fresh pericardium, consistent with the biochemical analysis, is largely depleted of the Gal antigen (Fig. 2D). Differential salt precipitation was used to compare the collagen composition of WT and GTKO pericardium (Fig. 2E). Precipitation with 1.7 M NaCl at neutral pH recovers mainly collagen III while collagen I and V are precipitated in 2.5 and 4.0 M NaCl respectively [21]. Both the WT and GTKO pericardium show typical [24] and similar collagen profiles at each salt concentration (Fig. 2E). There was no significant difference between WT and GTKO pericardium in the hydroxyproline estimate of total collagen content (Fig. 2F). Immunohistology of fresh WT and GTKO pericardium detects collagen I, III, and V staining in both tissues (Fig. 3) and is consistent with the differential salt precipitation of collagen in Fig. 2E.

### 3.2 Stress testing of fixed WT and GTKO pericardium

**Fig. 4:**
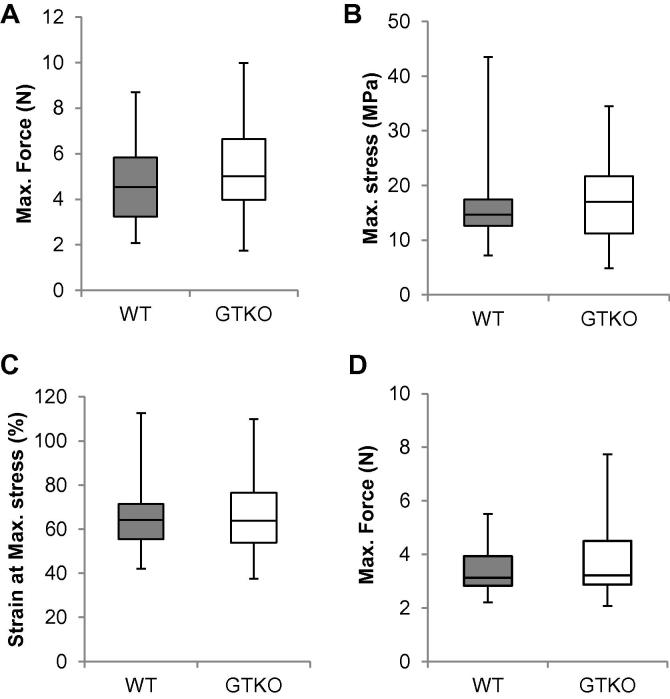
Box and Whisker presentation of uniaxial stress testing of glutaraldehyde fixed WT and GTKO pig pericardium (n = 32). A. Average maximum force (N) applied until failure (4.7 ± 1.8 N and 5.4 ± 2.2 N for WT and GTKO respectively). B. Average maximum stress (16.2 ± 7.5 MPa and 17.6 ± 7.5 MPa, for WT and GTKO respectively). C. Average strain at maximum stress (66.6 ± 18.4% and 66.3 ± 16.2%, for WT and GTKO respectively). D. Suture retention testing showing the maximum force required to tear a suture through the tissue (3.4 ± 0.9 N and 3.8 ± 1.5 N for WT and GTKO respectively, n = 15). No significance difference was detected between the tissues using a 2-way Student’s *t*-test. Average values are ± the standard deviation.

Uniaxial stress testing was used to compare the physical properties of fixed WT and GTKO pericardium. For these tests sample thickness was measured as 149.4 ± 32.2 μm and 158.1 ± 32.8 μm (mean ± standard deviation) for WT and GTKO pericardium which was not significantly different. Tensile strength recorded as 16.2 ± 7.5 MPa and 17.6 ± 7.5 MPa for WT and GTKO pericardium, respectively (Fig. 4A–C) and was not significantly different between the two tissues. The Young’s modulus was calculated at regions of low and high stiffness in the stress strain profile for each tissue type. In the low stiffness region, corresponding more closely to the forces exerted on valves in a BHV, Young’s modulus was 42.8 kPa and 48.6 kPa for WT and GTKO respectively (P value = 0.654). In the high stiffness region, young’s modulus was 386.8 kPa and 593.3 kPa for WT and GTKO respectively (P value = 0.052). In this region there was no significant difference but there was a trend for the GTKO tissue to be more resilient.

The forces measured in the suture retention tests to break the WT and GTKO pericardium specimen by pulling the VICRYL 5-0 were not significantly different, measuring as 3.4 ± 0.9 and 3.8 ± 1.5 for the WT and GTKO respectively (Fig. 4D).

## 4 Discussion

This study was designed to compare the composition and physical characteristics of WT and GTKO fixed porcine pericardium to test for physical equivalence. The mutation of the GGTA-1 gene in GTKO pigs only affects the terminal addition of alpha 1,3 linked galactose to lactosamine. This change would not be expected to substantially affect the physical properties of the tissue and we find no evidence that WT and GTKO pericardium exhibit a compositional change, other than the absence of the Gal antigen in GTKO pericardium, or any differences in the tensile stress properties of the fixed tissue. Similarly bovine pericardium and porcine swine intestine submucosa treated with alpha-galactosidase or in some instances decellularized by detergent extraction have also been reported to show elimination of the Gal antigen and physical equivalence to untreated tissue [25], [26], [27] further supporting our finding that the genetic elimination of Gal does not significantly alter pericardial tissue integrity.

There is growing interest in using Gal-free tissues to develop BHVs and other biomedical materials based on our research [15], [16], [20], [28] and others [17], [19], [29], [30], [31] indicating that the Gal antigen is present on current clinical BHVs and surgical meshes [31], [32], remains immunogenic and that binding of human anti-Gal antibody initiates a process leading to fixed tissue calcification which may promote SVD. This immune mechanism may especially contribute to age-dependent SVD where current anti-calcification processing has not been effective. There are a range of clinical reports where the combination of anti-Gal antibody and Gal antigen may have contributed to BHV loss including catastrophic valve failure in paediatric patients [33], BHV loss due to pork allergy [34], immune rejection [35] and even hyperacute rejection of a pericardial BHV [36]. Carbohydrate specific antibody mediated immune injury may also affect ABO mismatched homografts as there is a report of higher early reoperative rate in children with ABO incompatible cryopreserved homografts [37]. These experimental and clinical results highlight the significance of carbohydrate antigens which contrary to protein antigens are not effectively blocked by fixation and support the case that BHVs made from pig tissues devoid of the dominant xenogeneic glycan, Gal, are less immunogenic [20] and may result in more durable valves which resist anti-Gal antibody mediated calcification.

Pig pericardium is mainly composed of collagen and a range of associated proteins. The collagen component contains little of the Gal antigen which is mainly present in detergent soluble matrix associated glycoproteins (Fig. 1A and B). These Gal modified glycoproteins may be removed by detergent extraction from fresh pericardium, but resist extraction after glutaraldehyde fixation (Fig. 2C and D). The post-fixation retention of these proteins may account for the continued presence of the Gal antigen in current clinical BHVs which use detergent or ethanol based post-fixation anti-calcification processing.

Wild type and GTKO pig pericardium was collected from animals of approximately 6 months of age and had an average thickness of approximately 150 μm (Mean ± SEM, WT: 149.4 ± 5.7 μm and GTKO: 158.1 ± 5.8 μm). This is thinner than typical bovine pericardium and porcine heart valve leaflets. Thin tissue may be beneficial for use in percutaneous designs and may have beneficial effects on leaflet hydrodynamics but, may also have a negative impact on valve durability. Test valves based on these tissues will need to be developed to measure these parameters and to find an optimum for tissue thickness and durability.

Gal is not the only xenogeneic carbohydrate antigen present on commercial BHVs [38], [39], [40], but its high level of expression and the abundance of anti-Gal antibody in patients suggests it will have a disproportionate impact through this mechanism. Additional genetic modification which further reduces the antigenicity of pig tissue have been reported [40] suggesting that genetically modified antigen reduced porcine tissues can be a platform technology for glutaraldehyde fixed BHVs in the short term and in the long term for regenerative strategies to develop living tissue engineered heart valves and other devices.

## Disclosures

The authors do not have any conflict of interest to disclose.
